# Art (Pre)History: Ritual, Narrative and Visual Culture in Neolithic and Bronze Age Europe

**DOI:** 10.1007/s10816-020-09471-w

**Published:** 2020-07-20

**Authors:** John Robb

**Affiliations:** Department of Archaeology, University of Cambridge, Cambridge, UK

**Keywords:** Neolithic, Bronze Age, Iron Age, Europe, Prehistoric art, Rock art, Cave painting, Megalithic art, Visual culture, Narrative

## Abstract

Can we reconstruct how prehistoric people perceived things (their “ways of seeing” or visual culture)? This challenge is made more difficult by the traditional disciplinary assumptions built into prehistoric art studies, for instance focusing narrowly upon a single body of art in isolation. This paper proposes an alternative approach, using comparative study to reveal broad regional changes in visual culture. Although prehistoric art specialists rarely work comparatively, art historians are familiar with describing continent-wide general developments in visual culture and placing them in social context (for instance, the traditional broad-brush history from Classical to medieval to Renaissance systems of representation). This paper does the same for Neolithic (6000–2500 BC) vs. Bronze Age (2500–800 BC) and Iron Age (800 BC–Classical) rock and cave art from sites across Europe, uncovering broad patterns of change. The principal pattern is a shift from a Neolithic iconic art which uses heavily encoded imagery, often schematic geometric motifs, to a Bronze/Iron Age narrative art, which increasingly involves imagery of identifiable people, animals and objects. Moreover, there is also an increasing tendency for motifs to be associated in scenes rather than purely accumulative, and with contextual changes in how art is used—a movement from hidden places to more open or accessible places. Underlying all these changes is a shift in how rock and cave art was used, from citations reproducing ritual knowledge to composed arrays telling narratives of personhood.

## Big Histories of Art, Historic and Prehistoric

This paper attempts to look at prehistoric art in a way which is both new and old—entirely new for prehistoric art studies, but deeply familiar for visual culture in general. Imagine a range of the images commonly encompassed by the term “Western Art”. Without even thinking about it, you will almost certainly recognise what “period” they belong to. Their formal features—what subjects they depict, how “naturalistic” they are, how they use space and perspective and so on—place them stylistically within the canonical history of Western art: Classical, Medieval, Renaissance, neo-Classical, Impressionist, Modernist and so on. Moreover, these features, and others such as the place these images were originally designed to be seen in and the kinds of viewers they anticipate, helped form “ways of seeing” which were deeply intertwined with the pictures’ social context and historical moment. Visual culture changes historically as society changes; we can therefore write a big history of art. Indeed, though most art historians write specialist studies of particular artists and their oeuvres, all such studies assume and build upon a canonical big history of art; it would be impossible to understand Monet without understanding Impressionism or Michelangelo without Renaissance art.

Yet, a broad-brush, large-scale social history of prehistoric visual culture has never been attempted, or even contemplated. This is not for lack of material to study; Europe has 40,000 years of prehistoric imagery, in contrast with a mere 2800 years of historic art. It is not for lack of change to study; Spanish or French art made 30 or 40 millennia ago differs in every possible sense from late Iron Age art made 2000 years ago. It is not from lack of social change to contextualise such a history; the worlds of Palaeolithic foragers, Neolithic villagers and Bronze and Iron Age people differed from each other far more than the urban, stratified art worlds of medieval and modern people do. Instead, the reasons this project has never been attempted are disciplinary. One issue concerns the goal of interpretation; traditionally, archaeologists have focused overwhelmingly on what prehistoric art “meant” rather than on how it worked as visual or material culture. But above all, there is a relentless tradition of studying prehistoric art as single bodies of art rather than comparatively or analytically. The modal study of European prehistoric art is an authoritative descriptive analysis of a single corpus of material. Only rarely do analysts cross the boundaries between closely related corpora to talk about specific periods or areas. Even so, they rarely venture beyond synthesising homogeneous bodies such as “Ice Age Art” or “Celtic Art”. Larger scale efforts restrict themselves entirely to descriptive overviews (Sandars 1985). And, it must be said that prehistoric art specialists are often territorial about “their” bodies of art and defend them jealously against interlopers.

Thus, a big history of prehistoric European art has never been attempted, not because it is inherently impossible, or because it would not be worth doing, but simply because we have never imagined it as a possible goal. I argue that not only can we create such a history, we must do so. Looking at prehistoric art comparatively as visual culture can reveal things we would never understand from studies of individual corpora or periods, particularly about broad social change. Such a synthesis in no way threatens or supplants studies of individual bodies of art, any more than formulating the general ways in which Renaissance art differed from medieval art obviates a specialist study of Michelangelo or Leonardo. Instead, it provides an essential context for them.

## Prehistoric Art in Europe: Background and Materials

The “greatest hits” of European prehistoric art are familiar. The most famous images bracket prehistory. At the beginning, we have Ice Age caves such as Lascaux, Altamira and Chauvet, painted with vivid deer, bison, wild horses and lions; at the end, on the threshold of the Classical world, we have the beautiful swirling patterns of circles and spirals on “Celtic” metalwork. But such well-known images are only a tiny proportion of what is out there. If we define “prehistoric art” in the conventional archaeological sense of representative and decorative imagery, including cave art, rock art, tomb and megalithic art, statuary, stelae, figurines, figured objects of metal, clay and bone, and many other less common genres, there are hundreds of bodies of “art” known (Robb 2015). Much of it is deeply unimpressive, and the famous images are often famous simply because they look the most like modern “art” to us; for every Palaeolithic bison or fancy “Celtic” mirror, there are thousands of sketchily engraved bone fragments, lumpy, broken figurines or enigmatic circular rock carvings.

This analysis discusses a significant subset of prehistoric European art, Holocene pictures. Building upon the first statistical overview of prehistoric art in Europe (Robb 2015), it includes bodies of art from Russia to the Atlantic and from Scandinavia to the Mediterranean. Chronologically, it expressly does not discuss imagery made by Palaeolithic or Mesolithic foragers; this is a large body of art which works in different ways than later art, and including it would go well beyond what can be covered in a single article. The periods covered here include the Neolithic, Copper, Bronze and Iron Ages. The Neolithic begins with the transition to settled agricultural life; this happened between 6500 and 3900 BC in different areas of Europe. The later fourth and third millennia BC, known confusingly in different areas as the Middle Neolithic, the Late Neolithic, the Copper Age and the Early Bronze Age, are marked by dramatic social changes (see below). The Bronze Age *per se* begins around 2400 BC in most of Europe and develops continuously into the Iron Age between about 1000 and 800 BC. In many areas, the Iron Age ends with incorporation into historical Greek or Roman worlds, but in Northern Europe, Eastern Europe and Ireland, it continues organically into the early medieval period. In this article, I broadly contrast Neolithic art with Copper, Bronze and Iron Age art. This bears two caveats. First, while bringing together materials from across Europe allows us to identify a broad transition between the two periods, this provides a general heuristic rather than a mechanism driving changes in rigid lockstep. Nobody doubts that a general transition occurred from medieval to Renaissance art, but that does not mean this change occurred instantly at 1450 AD and synchronously everywhere in Europe; the same holds true for prehistoric art histories. Secondly, in keeping with related changes in prehistoric society, the transition in art varies regionally, occurring earlier south of the Alps and east, and later in Atlantic areas. Thus, for instance, Central Alpine rock art dating to the mid-3rd millennium BC embodies new themes and styles which continue on into the Bronze Age; at the same moment, in Atlantic France and Britain, rock art and megalithic art continued to work in long-standing Neolithic traditions which would not end there for several centuries more.

In this analysis, I discuss two-dimensional visual imagery, mostly from rock art, cave art and architectural art (designs on menhirs, megaliths, tombs and buildings). I exclude three-dimensional imagery such as statuary and figurines, as these are likely to involve different systems of visual representation. I also exclude small decorative motifs on pottery, bone and metalwork. These are ubiquitous in almost all prehistoric and historic material culture, and thus form a massive, unbounded dataset, and they give little purchase for a history of change; every period has bone objects decorated with small circles and pots decorated with geometric motifs. Moreover, prehistoric art occurs in “macro-traditions”, broad groupings of multiple, distinct but generically related bodies of material which share materials and conventions (Robb 2015: Table 3). This article reconstructs a sequence spanning the major macro-traditions of two-dimensional imagery in most of Holocene Europe. However, it intentionally excludes three well-known macro-traditions which follow different visual conventions. Interestingly, all are located at the margins of Europe. In northernmost Europe, from Alta in Norway to the White River in Russia, several bodies of rock art belong to a circum-Arctic forager tradition which has different themes and conventions from the mostly agricultural worlds of southern Scandinavian art. In Eastern Spain, Levantine cave art has an exceptionally varied and vivid mode of representation which has little in common with anything in Europe. Instead, it strongly resembles North African rock art traditions, and it is best regarded as a Saharan-derived tradition which somehow found its way north of Gibraltar. Finally, the elaborate frescoes in Mycenaean palaces, Minoan palaces and the Bronze Age houses of Akrotiri (Thera) portray unique themes in unique ways, possibly influenced by Near Eastern or Egyptian models.^1^ All three traditions are based upon iconographic repertories and depictive styles strikingly different from those found in the rest of contemporary Europe, much as Inuit art differs from contemporary Native American art in adjacent regions or ancient Egyptian paintings differ from Classical paintings, suggesting that they derive from different art historical trajectories.

**Fig. 1:**
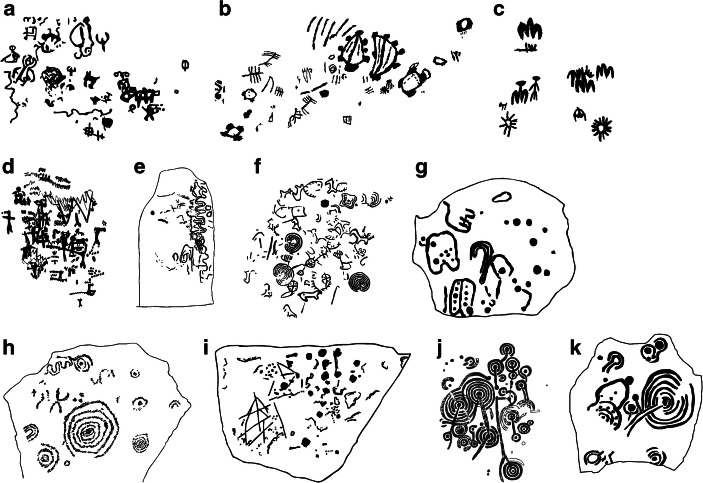
Some typical Neolithic panels. **a** Italian Neolithic cave art: Grotta Pazienza, Italy (redrawn after Gravina and Mattioli 2010: Fig. 7). **b** Italian Neolithic cave art: Porto Badisco, Italy (redrawn after Graziosi 1980: Plate 61). **c** Iberian schematic art: Peña Escrita, Fuencaliente, Spain (redrawn after Carmen Escobar Carrio, Wikimedia Commons). **d** Iberian schematic art: El Plato (redrawn after Sanchidrián 2005: Fig. 193). **e** Iberian megalithic art: Granja de Toninuela, Badajoz (redrawn after Shee Twohig 1981: Fig. 57). **f** Galician Atlantic Rock Art: Chan da Lagoa, Pontevedra, Spain (redrawn after Seoane Veiga 2007: Fig. 4). **g** Breton megalithic art: Le Lizou, Carnac (redrawn after Shee Twohig 1981: Fig. 137). **h** Irish megalithic art: Tara, Co. Meath (redrawn after Shee Twohig 1981: Fig. 245). **i** British megalithic art from a “domestic” context: Skara Brae, Orkney (redrawn after Shee Twohig 1981: Fig. 289). **j** British Neolithic rock art: Old Bewick, Northumberland (image: redrawn after Beckinsale archive plan/English Rock Art online). **k** Neolithic rock art, Scotland: Kirkdale House 4 (redrawn after 3d model by Scotland’s Rock Art, https://sketchfab.com/3d-models/kirkdale-house-4-456d69b988f449f8a5100bda1d9fcdfb)

**Fig. 2:**
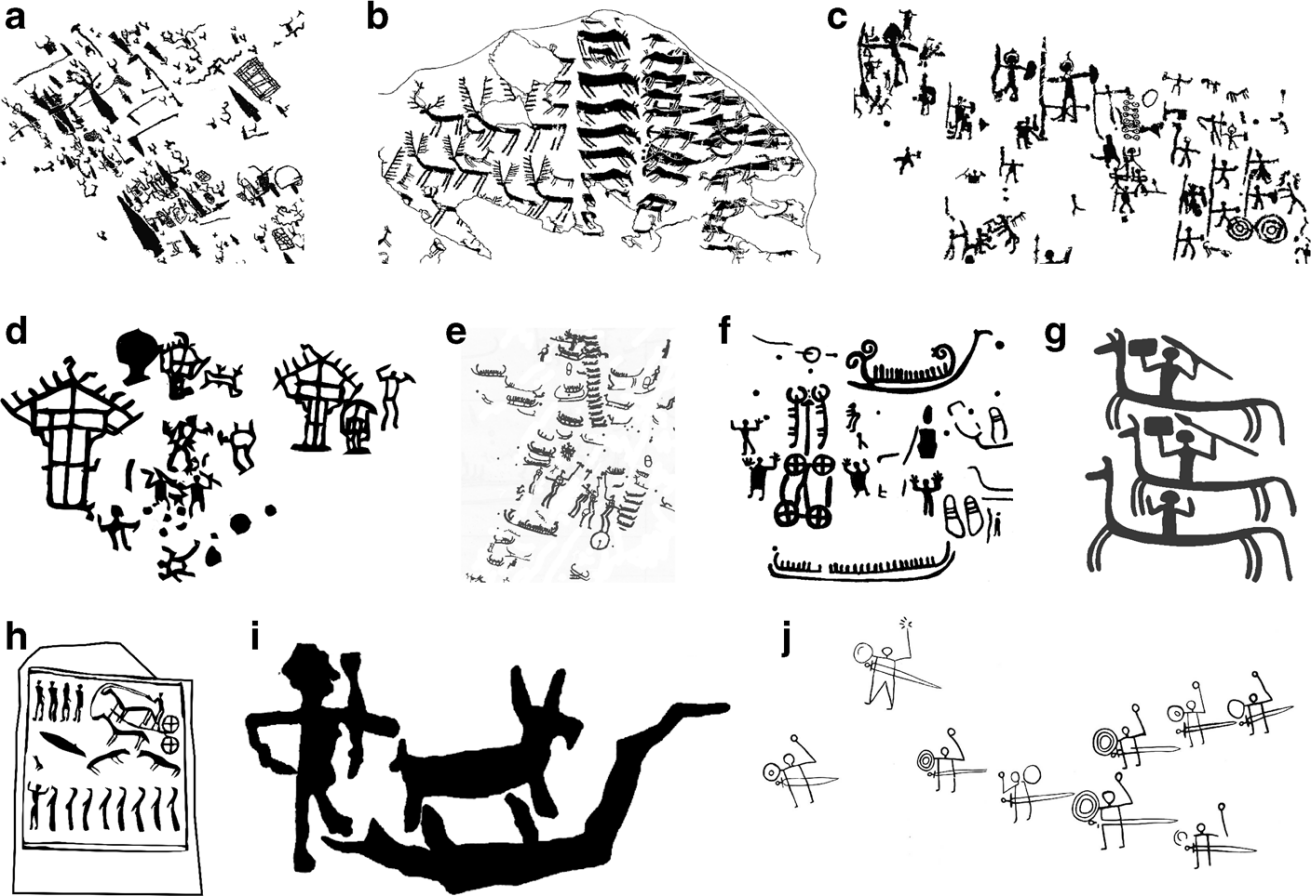
Some typical panels from 3rd millennium through 1st millennium BC (Copper Age, Bronze Age, Iron Age). **a** Mount Bego, France: daggers, halberds, oxen, ploughs and schematic motifs (Copper Age, 3rd millennium BC) (redrawn after de Lumley 1996: Fig. 271). **b** Alpine Copper Age art: arrays of deer, domesticated animals and weapons, Cemmo, Valcamonica, Italy, mid-third millennium BC (redrawn after *Parco Archeologico dei Massi di Cemmo*). **c** Central Alpine Iron Age art: Seradina, Valcamonica (redrawn after Marretta 2018: detail from site map). **d** Central Alpine Iron Age art: house and warriors, Seradina, Valcamonica, Italy (image: author). **e** Scandinavian Bronze Age art: boats, warriors and other anthropomorphs, animals, wheels. Tanum, Sweden (redrawn after Coles 2005: Fig. 162). **f** Scandinavian Bronze Age art: boats, carts and people (Askum, Sweden) (redrawn after Coles 2005: Fig. 200). **g** Iron Age rock art, Scandinavia: mounted warriors at Tanum, Sweden (drawing: Vicki Herring). **h** Megalithic art, Bronze Age: Kivik, Sweden (redrawn after Goldhahn 2009: Fig. 3). **i** Early Bronze Age rock carving of boat, animal and anthropomorph, Naxos, Greece (redrawn after Broodbank 2000: Fig. 23). **j** Thracian rock art (redrawn after Pivalaki 2016: Fig. 14.3)

Within these limits, this review attempts to be comprehensive, including about 20 major traditions of rock, cave and architectural art (Table 1, Figs. 1 and 2). I exclude some poorly dated sites such as Magura Cave (Bulgaria), but include some reasonably dated one-offs such as the Stonehenge rock carvings, Kivik (Sweden), German megalithic art and Levanzo Cave (Italy). I also make some reference to small, two-dimensional inset scenes on statue-stelae, metalwork and pottery, as these provide settings in which similar visual conventions come into play.

**Table 1:** Principal traditions of two-dimensional imagery in Europe, Neolithic through Iron Age, discussed in this article. Note that for most, no single major scholarly publication is available, and information is principally based upon site visits and internet material for key sites

Body of art	Location	Dates	Details
Central Mediterranean Neolithic cave paintings	Southern and Central Italy	5th–4th millennia BC	In caves, some very difficult to access. Mostly geometric and abstract figures, with a few anthropomorphs and animals. Porto Badisco Cave and other, less extensive, poorly known sites.
Iberian “schematic” and “macro-schematic” art	Spain and Portugal	5th–3th millennia BC	Mostly Neolithic and Copper Age. Painted motifs in caves and rock shelters. Dominated by highly schematised motifs, but including recognisable anthropomorphs, animals, and a range of other motifs; things that look partially anthropomorphic and partially unrecognisable are common.
Iberian megalithic art	Western Spain and Portugal	Later 5th-3rd millennia BC	Mixture of painted and carved motifs in megalithic tombs; almost exclusively geometric designs.
French megalithic art	Brittany and some adjacent areas of Normandy	Later 5th-3rd millennia BC	Carved and painted (Primitiva Bueno Ramírez et al. 2015) motifs in megalithic tombs and on menhirs; repetitive schematic motifs, potentially including axes and ox-horns but also including many unidentifiable ones. A few potential cosmological anthropomorphs (Shee Twohig 1981).
Maltese megalithic art	Malta	Mid-4th to mid-3rd millennia BC	Designs on “temples” such as Tarxien and hypogea such as Hal Saflieni. Mostly geometric designs (branching spirals); a few animals. (Possible boat motifs may be later additions).
German megalithic art	Germany	4th millennium BC	One or two megalithic tombs (*e.g.* Gröhlitz) with incised designs, including a bow
Sardinian tomb art	Sardinia	4th millennium BC	Decorations in “*domus de janas*” tombs. Motifs include bucrania, architectural elements, and geometric/schematic designs; anthropomorphs rare and may be later.
Mount Bego rock carvings	France	4th-3rd millennia BC	Open-air rock carvings in high-altitude Maritime Alps; mixture of abstract motifs, a few anthropomorphs and ploughs, and many repetitive oxen and weapon motifs.
Alpine Copper Age rock art	Italy	4th-3rd millennia BC	Sporadic small open-air sites throughout Central and Western Italian Alps, mostly with cup marks.
Galician rock art (“Atlantic rock art”)	Northwestern Iberia	Late Neolithic–Early Bronze Age, 4th-3rd millennia BC	On open-air rock surfaces. Dominated by geometric motifs, especially cup and ring motifs; some animals and weapons
British Neolithic rock art	Northern England and Scotland	3rd millennia BC	On open-air rock surfaces. Dominated by cup-and-ring motifs, with some spirals and other geometric motifs; very few non-geometric motifs.
Irish megalithic art	Ireland	3rd millennia BC	Often elaborate decoration of tombs such as Newgrange, Knock, Carrowkeel, etc. Exclusively geometric designs.
British megalithic art	Northern Britain	4th-3rd millennia BC	Schematic carvings on structures, exclusively geometric designs, often in concealed locations (Orkney).
Valcamonica Copper Age rock art	Central Alpine Italy	3rd millennia BC	Rock carvings in Valcamonica on open-air boulder masses such as Cemmo; similar motifs are found on statue-menhirs and statue-stelae in the same area. Motifs include daggers and other weapons, wild and domestic animals, ploughs, anthropomorphs and cosmological motifs.
Paris Basin tomb art	France	4th-3rd millennia BC	A few schematic motifs of breasts and necklaces in megalithic tombs.
Aegean Early Bronze Age rock art	Greece	3rd millennium BC	A few open air sites with carved motifs of boats.
Stonehenge	England	Late 3rd-early 2nd millennia BC	Daggers and axes carved on one stone at Stonehenge, an arly Bronze Age addition to the Neolithic site.
Kivik	Southern Sweden	Mid-2nd millennium BC	Megalithic tomb with rock-art style decoration (motifs of weapons, chariot, horns, anthropomorphs, wheels).
*Tombe di giganti*	Sardinia	Late 3rd-early 2nd millennia BC	Megalithic tombs with anthropomorphic facades.
Castelluccio	Sicily	Late 3rd-early 2nd millennia BC	Rock cut tomb with carved façade.
Northern Greek Iron Age rock art	Thrace	1st millennium BC	Open-air rock carvings of warriors, animals and some schematic motifs
Southern Scandinavia Bronze/Iron Age rock art	Southern Norway, Sweden; Denmark	Later 2nd–1st millennia BC	Open-air rock carvings. Most mid to late Bronze Age, some Iron Age. Widespread across southern Scandinavia. Common motifs include humans, boats, horses and other animals; a subset of motifs may refer to a cosmological narrative (Kaul 1998; Bradley 2006). Often occurring in thematic groups or scenes.
Central Alpine Iron Age rock art	Central Alpine Italy	Later 2nd–1st millennia BC	Open-air rock carvings. Valcamonica and adjacent areas such as Valtellina. A few later Bronze Age; mostly Iron Age. Great numbers of petroglyphs include wide range of motifs, dominated by humans, animals (deer and domestic animals), weapons, ploughs, houses, unidentified objects, often arranged in groups or scenes. Some geometric and schematic motifs.

## Concepts and Methods

The term “art” is used in this article in the conventional archaeological sense of “representational and decorated objects” such as figurines, sculptures, paintings and so on; I explicitly disavow any notion that these objects must have been meant principally for aesthetic enjoyment or to express discursive meanings and creative mastery of the people who made them. Indeed, in Gell’s (1998) sense of art as a social technology, they accomplished many tasks, from presencing spiritual beings to asserting social power; if a comparison with modern things is warranted, many of them would be better understood not as “art” but as interior decoration, narration devices, outlets for spiritual power or even medical technologies. Nevertheless, just as visual culture encompasses not only fine art but also advertising posters, wallpaper and family photographs, they embody the visual conventions and meanings of their times. There are several strands of visual culture research from which we can draw inspiration. In art history, Gombrich (1962) pioneered exploring styles such as naturalism and visual systems such as perspective. Half a century on, as Mitchell’s provocative question “What do pictures want?” highlights how the interaction between people and images is complex and reciprocal (Mitchell 2006; Belting 2011; Moxey 2008; Mitchell 1998). “Ways of seeing” (Berger 1972) are grounded in how viewers interact with images, often unconsciously; they include not only conventional systems such as perspective, but also habitual themes, scenes and internalised reactions about the act of viewing these. These also reflect conceptions of the shape of space (Summers 2003). Forms of vision are historically specific and are both attuned to and constructed by art (as in the concept of the “period eye” (Baxandall 1972; Alpers 1984)). History may be a succession of forms of visuality (Davis 2011). Converging with this, anthropologists have argued that aesthetic senses are culturally specific (Coote and Shelton 1992; Heyd 2012) and may be enmeshed with social reflexes (Gell 1998). Moreover, many examples show how aesthetic reflexes relate to ontological and cosmological presuppositions. For instance, pattern in Aboriginal art embodies concepts of ancestral spiritual power (Morphy 1992), while indigenous Andean metalwork techniques were inseparable from concepts of material and purity (Lechtman 1984). Similarly, in animistic traditions, an object can be both a crafted object and a spirit (Bray 2009); in our own tradition, a theological divide between matter and spirit underlies an understanding of “images” as representations of reality rather than reality itself.

Critically, modes of vision are enmeshed with identities and power relations. One example is the gendered male gaze, at the heart of much Western art since Classical times (Koloski-Ostrow and Lyons 1997; Berger 1972). Another is colonial mode of vision, embedded in practices of surveillance, mapping, photography and the racialised gaze of “natives” (Smith 1998). For this research, the key point is that modes of vision are historically specific and fundamentally embedded in systems of orientation, cultural values and sociopolitical relations. Moreover, they are signalled or triggered through material codes such as choice of medium, framing, composition and style of execution.

This is heterogeneous ground to summarise briefly, but all of these establish useful foundational principles:

- The act of seeing is not a neutral or universal act, but is constructed within a given social and historical context;
- There is a fundamental link between social contexts, especially relations of power, and the habituated reflexes of the viewer;
- There is a fundamental link between the habituated reflexes of the viewer and the material, thematic and stylistic characteristics of visual culture (using visual style in the sense of a way of doing things, akin to aesthetic style or technological style, and potentially reproduced through formal characteristics). Visual culture works with and reproduces these reflexes, and indeed in some cases provides explicit clues to how it should be interpreted.

In prehistory, power took radically different forms than it did in the urban, class-stratified societies visual culture studies typically deal with, but this general line of inquiry—examining art to see what it tells us about the act of seeing—is worth extending to prehistoric worlds. Archaeologists have rarely adopted a visual culture approach to prehistoric art, but some pioneering efforts have yielded important insights (Bradley 2009; Jones et al. 2011; Skeates 2005; Robin 2009; García Sanjuán et al. 2006; Wells 2012; Garrow and Gosden 2012; Helskog and Olsen 1995; Fredell et al. 2010; Fahlander 2012; Cochrane and Jones 2012; Primitiva Bueno Ramírez and Bahn 2015). It has often been pointed out how art made creative use of the physical features of its settings; for instance, Palaeolithic art sometimes utilises the 3-dimensional topography of cave walls to define imagery, and Swedish Bronze Age rock art may have used water running naturally over surfaces to appear animated. Bailey (2005) has discussed how figurines act psychologically upon people handling them. Gosden, Garrow and colleagues (Garrow and Gosden 2012; Gosden et al. 2008) have applied Gell’s concept of “technologies of enchantment” to Celtic art, noting its capacity for drawing in and bewildering the viewer. Wells (2012) has identified aesthetic patterns across genres of material culture in Iron Age Central Europe, relating them to social changes in the mid-late 1st millennium BC. Most relevant for this study, Jones (Jones 2012b, 2012a; Jones et al. 2011) has interpreted British Neolithic rock art and material culture as reflecting an animated world view, and Ranta et al. (2019) have used art theory to identify narrative characteristics in Bronze Age Scandinavian rock art. We return to these studies below.

While prehistorians can take inspiration from modern visual culture studies, for methods, we are on our own. Our data are necessarily coarse-grained, and the social context we can assign them often is as well. Moreover, we necessarily work with “formal” methods deriving interpretive clues from the material itself rather than “informed” methods which place it within a long-standing ethnographically known tradition, as in Australia, South Africa and the Americas (Tacon and Chippindale 1998). Here, I take the simplest possible approach, asking straightforward questions about each body of art:

- Where is it located? What kind of context was this, what kind of people had access to it and what did they do there?
- Does it depict things we can identify? How do these tell us about its social context and meanings? How are things depicted? Is it part of a coherent visual strategy?
- How are the art’s motifs arranged? Are images grouped spatially or related thematically? Is there an overall spatial or thematic order, or are they random accumulations of independent motifs?

As a first attempt at this project, these questions are evaluated here in a broad, qualitative way, by characterising tendencies within an entire corpus. This is what is possible; for no substantial corpus of prehistoric art would the available data allow rigorous statistical analysis, and even less possible is comparative analysis using data created by applying similar methods to disparate corpora. Characterisation is based upon both publications and in many cases personal observations. Such an analysis suffices to reveal some preliminary broad patterns.

## Three Key Trends in Prehistoric Art

### Location: Bringing Art into Society

Where was art located? Neolithic two-dimensional art is found in three distinct kinds of places: megalithic tombs, caves and open-air rock outcrops. Megalithic art, by definition, is found in specially constructed places not frequented as part of daily experience. Where exactly it is located within tombs varies, but generally carved and painted designs often emphasise specific zones such as portals (as in Sardinia and Ireland (Robin 2009, 2016, 2010), or the deeper, more inner area of tombs (as in Brittany (Shee Twohig 1981)). In Malta and Sardinia, tomb art also defines architectural elements, effectively helping create the tomb as a special place; the same may be true for the heavily decorated kerbstones at Newgrange (Ireland). At Gavrinis (France), the tomb’s internal walls were covered with complex designs. Whether or not tomb art was related to altered states of consciousness (Lewis-Williams and Pearce 2005; Lewis-Williams and Dowson 1993), it is clear that it formed part of special places, probably imbued with some form of supernatural power.

Neolithic cave art is found principally in Italy and Spain. The most extensively painted Italian site is Porto Badisco Cave, near the tip of the Salentine peninsula in south-eastern Italy (Graziosi 1980). Porto Badisco is a deep karstic cave whose narrow, twisting galleries are difficult to negotiate. Whitehouse’s spatial analysis of the cave showed that “representational” motifs are found closer to the entrance, while deeper zones of the cave contained only highly schematised motifs; Whitehouse interpreted this as evidence that people penetrating deeper into the cave were increasingly initiated into secret knowledge (Whitehouse 1992). Deep caves were often used as loci for special ritual practices, perhaps because of their otherworldly quality (Whitehouse 1992). Other Italian decorated caves are shallower, but often in locations difficult to access (such as on steep rocky cliffs below the crest of the Gargano massif (Gravina and Mattioli 2010)), and probably not used for principal habitations. The same is true for many Iberian painted caves and rock shelters (for instance, accessing some of the Rio Vero caves required scaling cliffs). Some Schematic Art caves may have been territorial markers located between sites (Lancharro Gutiérrez and Bueno Ramírez 2017).

Open-air Neolithic rock art occurs principally in Galicia and northern Britain. Whether people lived at or near sites is hard to assess, as few sites have been investigated archaeologically. But in areas such as northern Britain, some rock art is found on high moors, hilltops or slopes and most petroglyph sites may have no particular relation to settlement. Contemporary with these in the 4th-3rd millennia (and transitional to the changes discussed below) is the Copper Age art of Mount Bego, France. Like contemporary rock art in Valcamonica, this includes a mixture of third-millennium motifs such as weapons, oxen and ploughing, but also some figures which may represent cosmological anthropomorphs and symbols. The Mount Bego art is found at very high altitudes (above 2000 m), well above farmable zones and probably frequented only seasonally by special purpose groups.

Later art contrasts strongly with this. Art was no longer especially associated either with the dead, or with remote or difficult to access places. Nor were caves chosen as a location to place images; there is almost no cave art after the third millennium BC. Instead, art moves out into frequented zones. In Thrace, rock art occurs around the edges of lowland basins (Pivalaki 2016). The two mega-concentrations of later Bronze Age and Iron Age rock art are in southern Scandinavia and the Central Alps, both well-known areas where hundreds of thousands of petroglyphs are known. In southern Scandinavia, in areas such as Tanum (Bohuslän) and elsewhere, carved rock outcrops occur in lowland valleys. These are readily accessible today, and in prehistory, many were along navigable inlets, lakes or streams which would have been communication routes and loci of settlement. In the Central Alps, according to GIS study of the spatial distribution of Valcamonica’s rock art (C. Alexander, pers. comm.), major rock art sites such as Naquane and Seradina are located just above the valley bottom where habitations and cultivated land were located, within 200–400 m of settlements, in areas within easy view and possibly audible range of communities. In both areas, rock art is part of a familiar, frequented landscape.

### From Abstract Icons to Recognisable Motifs: Schematism as a Visual Strategy

“What is it a picture of?” This obvious question is very revealing, but not as we ordinarily think of it. Here, let us begin with a simple generalisation. In Neolithic imagery (Fig. 1), we can recognise relatively few referents confidently. Some bodies of imagery contain only abstract or geometric shapes: cup marks, circles, spirals, labyrinths, lines, crosses, zig-zags and so on. This is true for British and Irish rock art and megalithic art. Other bodies of images contain these signs, but also some recognisable anthropomorphs (images which look human, but which may represent human-ish beings rather than people *per se*), or perhaps some animals, usually deer. This typifies Italian Neolithic cave art and Iberian megalithic art. Breton rock art contains highly repetitive, highly stylised motifs, but almost always connoting a referent we can only guess at; triangles may or may not represent axe heads, a doubly curved line may represent ox horns, and so on. There are also stereotyped motifs such as the “buckler” and the “Mané axe” (Shee Twohig 1981) whose referent is entirely a matter of conjecture. As one gets into the third millennium BC, the range of recognisable things increases. Beyond a range of geometric signs, Iberian “schematic” art and “macro-schematic” art includes anthropomorphs, animals, and a range of less frequent motifs possibly representing things such as boats. Galician rock art includes animals and weapons as well as cup marks, rings and other schematic motifs. At Mount Bego, the repertory includes common Copper Age iconography such as weapons, oxen and ploughs (de Lumley 1996; Huet 2017), and in Valcamonica, Copper Age imagery includes daggers, deer and anthropomorphs. Both Iberian “schematic” art and Mount Bego also contain stylised, abstract motifs and enigmatic motifs that look human-ish but may be supernatural beings and/or people wearing ritual costumes, and Valcamonica motifs include cosmological solar signs and spirals.

In later periods (Fig. 2), we see more and more things that we recognise. Southern Scandinavian rock art contains lots of people, often gendered with anatomical details or hair styles, often with stylistic differentiation or distinguishing details. People are often shown not merely standing in a stereotyped frontal pose, as in earlier periods, but in a wide range of action poses. It contains lots of boats, sometimes with specific details of construction that allow seriation or phasing. It contains identifiable deer, horses, cattle, pigs and other animals. It contains several distinct, recognisable kinds of weapons—swords, spears, bows and shields. And there are many other, less common but still identifiable motifs such as footprints. Many of these motifs mirror themes known in contemporary Hallstatt art (for instance, warriors, water birds, people jumping acrobatically). Others may depict in quite concrete terms specific narratives we can now only reconstruct, but which must have been well-known to contemporary audiences; the grouping of boats, horses, serpents and wheels may refer to a specific cosmological narrative (Kaul 2005, 1998). Bronze and Iron Age Alpine rock art parallels these trends closely. At Valcamonica and Valtellina, there remain schematic images, sometimes simple ones such as the so-called “Camunian rose”, sometimes complex ones such as the “maps” at Bedolina. But there are many people, sometimes distinguished by headgear or costumes and often shown doing things rather than in the static frontal position of earlier petroglyphs. There are multiple kinds of weapons and animals. Other objects depicted include ploughs and houses, granaries, footprints and even musical instruments. Even when we cannot identify a motif, we can still have confidence that it represents a specific object rather than (say) an abstract concept or quality or supernatural force; the so-called “palletta” motif at Naquane is an example. At the eastern end of Europe, sporadic examples of Aegean Bronze Age rock art show boats, and Thracian rock art again similarly is dominated by recognisable imagery of warriors and horses (Pivalaki 2016). Overall, compared to earlier periods, later imagery is much more readily comprehensible. It still contains “abstract”, “non-representational” or highly schematised motifs, but the general subject of the art is a lot clearer and the number of things we can identify is much higher.

**Fig. 3:**
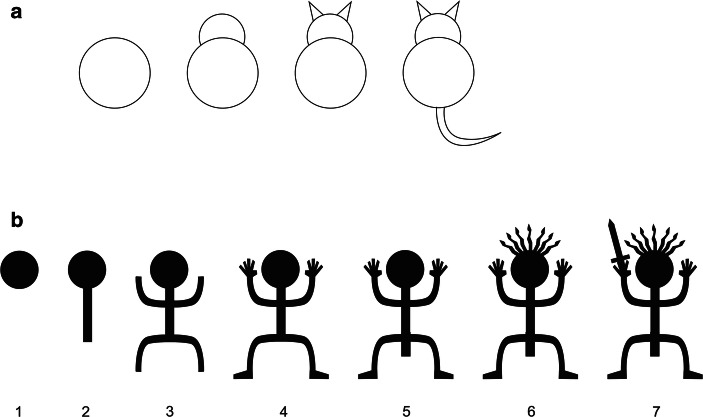
Schematism and detail as visual strategies. Each detail added specifies a specific interpretation and precludes other possible polysemic interpretations. **a** Cat (image: redrawn after Gombrich 1962: Fig. 3). **b** A rock-art style anthropomorph (image: author)

How should we understand this shift? Archaeologists often characterise motifs as “representational” or “abstract”, but this distinction is debatable. An “abstract” motif almost certainly had little to do with abstract meanings as in modern art; instead, it may well have denoted something quite concrete and specific which we lack the context to identify. However, nor is it useful to simply blame lack of recognisability on our modern ignorance of an image’s context and give up on interpretation. Instead, it is more useful to focus on schematism as a visual strategy. What we experience as recognisability or non-recognisability reflects a representational choice, how much information is encoded in an image. This was first pointed out by Gombrich (1962) in his discussion of “naturalism” in art. In Gombrich’s example, suppose you want to depict a cat (Fig. 3a). You could represent a cat by a simple circle; for those who already know that circles represent cats in this context, this would be an adequate representation. But a circle can denote many other things. As you add additional detail, it increasingly precludes other interpretations and restricts interpretation to a cat. With further detail—its stripes or spots, its position—you might be able to indicate a specific cat, or a cat in a particular mood or situation. The same logic holds for an anthropomorph (Fig. 3b). If understood contextually as such, a simple circle or line may simply indicate the presence or absence of a human being. By the time we add several levels of detail, it may show a gendered person of a particular category, activity or state of mind, perhaps even a specific individual.

Schematism, therefore, is not simply a matter of style; it is a visual strategy prompting specific ways of reading an image. Schematic images are useful when you want a simple, broadly applicable categorical identification (as with bathroom doors, brand logos, and icons identifying social groups). It can be useful when you want to invoke or summarise complex or immaterial referents compactly (a cross summarises Christianity as a complex set of beliefs). It can also be useful for depicting things that can have meanings on multiple or shifting levels, or that mean different things to different audiences. For example, some Australian aborigine groups represent narratives through highly schematic, polysemic iconography; while each sign can have multiple referents, their combination restricts possible meanings and constructs a specific interpretation (Munn 1973). This may be particularly appropriate in a cosmology that can have multiple layers of reality which reference each other. In contrast, adding detail constrains the potential interpretations you can apply to an image. It restricts polysemy and layered or creative interpretation. Adding detail allows nuanced categories, avoids ambiguity and guides complex or specific interpretation, as in the anthropomorph example (Fig. 3b). It also demands less of viewers, by presenting more of the context and interpretation rather than requiring them to supply it. There are many familiar examples of how images vary in schematism or detail according to how they are intended to be interpreted or to act upon viewers. The simple glyph indicating a male or female public bathroom connotes a simple category, very much in rock art style. A wiring diagram or subway map eliminates some information (for instance, about actual distances and directions) in order to communicate other information (topological relationships between nodes). At an intermediate level, mixing schematism and naturalism, saints in medieval art are often shown as generic figures distinguished by a single symbolic diacritic—the arrows for St. Sebastian, the skull for St. Jerome, the wheel for St. Catherine. Schematism as a rhetorical strategy is exemplified by the kind of semi-naturalism found in both Classical sculpture and modern clothing catalogues (Robb and Harris 2013: Chapter 4). In this, people are shown in “naturalistic” detail, but without individualising features; the effect is to draw the viewers into a generic lifestyle they can identify with and aspire to. At the most detailed end of the scale, the photograph on a passport has enough detail to be matched to a specific individual.

As we go from the Neolithic to the Iron Age, therefore, we are not really seeing a shift from “abstract” to “representational” art. Instead, we see a shift in visual strategy, from highly schematised images to more detailed and explicit images. In terms of what acts of interpretation they required of the viewer, it is a shift from more highly encoded, contextually defined meanings to meanings which are more visually transparent and tied to single concrete referents—even to the point where uninformed viewers such as ourselves can identify them.

### From Single Motifs to Composed Scenes

If you look at a panel of rock art, cave art or tomb art, do the motifs touch each other? Are they in some evident spatial arrangement? Do thematic relations among them group them into sets of related objects?

Neolithic visual culture rarely includes composed scenes. Motifs are typically jumbled on a surface in a way which suggests that they were added one by one. Moreover, they do not often form recurrent sets. To the extent that we can deduce a spatial logic, it is minimal: motifs usually avoid directly intersecting one another. The rest appears randomised and accumulative. To take an example, Porto Badisco Cave has one or two famous (and often reproduced) panels that form scenes, but all the rest of the cave’s art consists of apparently random scatters of motifs, and this is true of the rest of Italian Neolithic cave art. Throughout Neolithic art, the only consistent spatial arrangements occur in Sardinian, Breton and Irish tomb art, where there are some weak but consistent patterns of how different motifs are distributed within tombs to define architectural spaces (Robin 2009, 2016, 2010). These occur at the level of the entire site; panels themselves usually appear unordered. The individual motif may have been the unit of citation or inscription, and the act of making a motif more significant than the result.

From the 3rd millennium BC onwards, tendencies to spatial patterning in rock art appear. A simple test is to ask how many motifs touch another motif, are grouped thematically with other motifs, or occur in clear spatial arrangements such as arrays. Third-millennium sites in the Alps already show these features. At Valcamonica, Copper Age rock art contains repetitive arrays of animals and weapons. At Mount Bego, oxen motifs are shown yoked in pairs, occasionally with a plough. This tendency towards spatial order accentuates in the later Bronze and Iron Age. In southern Scandinavia, the Central Alps, and Thrace, rock art is full of arrays, connected motifs and even scenes. Boats and animals form lines. A human figure holds a sword, shield, bow or spear. A horse has a rider on its back. A boat motif is combined with line-strokes representing its crew members. People face each other, fighting. A human drives a team of animals. A hunter chases deer, helped by dogs. Such composed groups appear in almost every rock art panel. There may also be less obvious thematic groupings, as with the Scandinavian sun-boat-cart-snake-horse narrative (Kaul 1998). In some ways, this is a logical extension of the trend discussed above, of adding detail to motifs to characterise specific situations more explicitly: at some point the informational burden becomes too great to communicate through a single motif. Increasingly, the unit of visual communication is not a single motif, but an interconnected group of motifs.

**Fig. 4:**
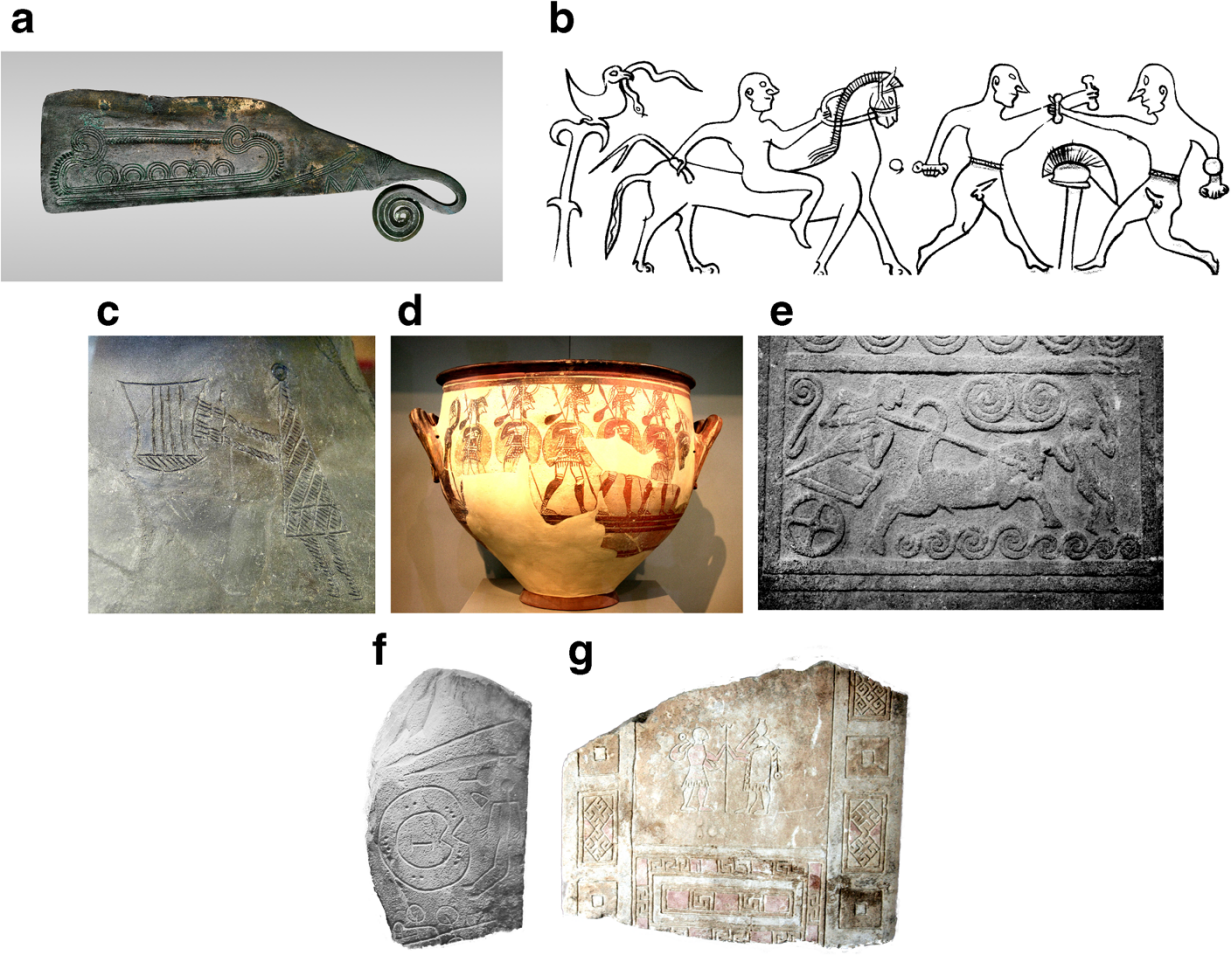
Bronze-Iron Age inset scenes in other media. **a** Inset scene of boats, razor, Bronze Age Denmark (National Museum, Copenhagen) (image: John Lee, Wikimedia Commons under CC licence). **b** Situla art: inset scene of athletes on Iron Age metal plaque, Vienna, Natural History Museum (image: redrawn after National History Museum, Vienna). **c** Inset scene of lyre player, Iron Age Sopron pottery, Natural History Museum, Vienna (image: Wolfgang Sauber, Wikimedia Commons under CC licence). **d** Inset scene of warriors on Mycenaean pottery, mid-2nd millennium BC (National Archaeological Museum, Athens)(image: author). **e** Inset scene of charioteer on stela, Mycenae, Greece (mid-2nd millennium BC) (National Archaeological Museum, Athens) (image: author). **f** Biographical imagery of warrior on funerary statue-stela, Caceres, Spain (*Museo Arqueologico Nacional*, Madrid) (image: author). **g** Inset mythological scene, statue-stela, Puglia, Italy (*Museo Nazionale di Manfredonia*; image: author)

Interestingly, it is at the same time that composed scenes appear in other media, usually as small inset scenes (Fig. 4). Prehistoric Europeans had made pottery since the 5th or 6th millennium BC, and pottery provides an ideal surface for expression; Neolithic and Bronze Age pottery is often highly decorated. But, except for a few rare anthropomorphic or animal images, it is entirely schematic. Recognisable images on pottery increase from the third millennium BC onwards. Only in the Iron Age do actual composed scenes of associated imagery occur, as on the Sopron Iron Age vessels from Austria (Rebay-Salisbury 2016). In metalwork, motifs grouped into composed scenes occur from the later Bronze Age, as in Danish razor art. Iron Age “situla art” from the upper Adriatic shows elaborate pictures of social life and mythology; the Gundestrup Cauldron is covered in floridly mythological scenes. Statue-stelae are large stone anthropomorphic sculptures; the genre begins closely linked to megalithic ritual sites in the 3rd millennium BC and evolves into individual commemorative markers by the 1st millennium BC (Robb 2009). They are sometimes decorated with inset scenes. In early ones, at Valcamonica and nearby regions such as Lago di Garda, motifs more or less randomly accumulate on the statue-stela’s surface. In later ones, such as Iberian Bronze Age examples, they form thematic groups (for example, a spear, shield and horse as part of a male biographical narrative). Taking this further, the Iron Age Daunian stelae of south-eastern Italy (mid-1st millennium BC) sometimes have quite detailed inset scenes. It is easy to miss the significance of such composed, narrative scenes on pottery, metalwork and statues because they have been a staple of Western art ever since the Iron Age. But the point is not only that they occur increasingly from the 2nd millennium BC onwards, but also that they virtually never occur before this period. They confirm the thesis of a general reorganisation of visual culture in later prehistory.

Besides parallels in other media, the Bronze Age reorganisation of vision has strong echoes in burial practice. Neolithic deathways varied, but they often involved disassembling the body into fragments and dispersing it among landscapes or sites. There is little attempt to preserve or portray a recognisable individual; the body is unbundled into cosmological citations, perhaps much as art motifs are. Beginning in the 3rd millennium BC, it is increasingly common to present the dead person as a composed individual defined by key objects tying him or her to stable story lines of social prestige. This echoes the emergence of individuals in art, not only in themes such as gendered action, but also in the idea of the body as a central, constant and recognisable unit giving continuity to social presence. Such close parallels are not surprising, given that burial itself is a form of visual culture, and Bronze and Iron Age burial in particular was often aimed at composing a memory-fixing tableau of the social persona in death.

## Visual Culture and Society

Compare the imagery in Figures 1 and 2. This overview has highlighted three basic trends:

1. Between the Neolithic and the Iron Age, art increasingly comes out in the open, moving from caves, tombs and sometimes remote areas to well-frequented landscapes.
2. Between the Neolithic and the Iron Age, images become less schematic, increasingly detailed, and more transparently recognisable.
3. The unit of visual communication shifts increasingly from single motifs to arranged groups of motifs, often in action poses or scenes.

These trends are not visible in any single body of material, since virtually all bodies of prehistoric art are restricted to a single period and thus cannot reveal long-term change; moreover, the available corpora are patchily distributed and provide geographically scattered windows into what must have been a continuous, widespread process. And these are not black and white changes. Neolithic art contains some recognisable motifs and composed scenes; there are plenty of schematic and unarranged motifs in the Bronze and Iron Ages. But if we view all prehistoric pictures collectively, as a kind of single, diverse super-corpus attesting prehistoric visual culture, the outlines of a big history of prehistoric art begin to emerge.

### The Neolithic: “Art” as Cosmological Knowledge and Action

To move from describing this change to interpreting it, the key is asking how people interacted with imagery. Fortunately, the art’s context and structure themselves contain clues to this. People often encountered Neolithic art in unusual or out of the way places such as tombs or inaccessible caves, not everyday places; many of the sites are what might be broadly characterised as specialised ritual settings. When people encountered it, its highly schematised mode of representation demanded contextual knowledge; understanding it correctly required them to already know which of the many things a circle or wavy line could connote was meant in a particular context. The same is true on the scale of a whole panel of motifs. Except in a few cases in which imagery was used to customise architectural areas of built structures, the viewer is not presented with an overall pattern to recognise. The grain of the assemblage is that of episodic single acts accumulating on a surface which was important for some reason.

This raises the question of the ontology of “art”. Perhaps because we understand imagery representationally ourselves, we tend to see something like a carved circle or painted line semiotically, as a communicational medium which represents something to a viewer. Indeed, our reflex to ask about the “meaning” of imagery inherently invokes a representational paradigm in which signification is distinct from the thing itself, which merely acts as a vehicle. But, as colleagues have pointed out (Jones 2017), representation may be the wrong way to understand Neolithic art. A representational paradigm implies that the material signifier is arbitrary or unimportant, but Neolithic art was demonstrably concerned with material, light and location (Cochrane et al. 2014; Jones 2012a; Jones et al. 2011). The minimalism of Neolithic art suggests that, once something is specified enough to define what it is, there is actually very little further concern with representation. Similarly, a semiotic framework would imply that being seen was the important thing; in its size, medium or landscape position, Neolithic art often shows little concern for visibility, and the act of making a mark or its simple existence may have been the important thing. Neolithic art may have been not about representing reality but acting upon it, as a material operation on the world. Both Australian and South African rock art are known to sometimes have acted as interventions in a spirit world, as ways of contacting or channelling cosmological forces. To take an example, much of British Neolithic rock art consists simply of circles and connective lines (Fig. 1). If you imagine an earth whose underlying stones contain cosmological power, then such interventions may have been a way of creating points of contact with the inner world of stones, perhaps similar to the way in which, in South African rock art, rock surfaces acted as the interface between human and spirit worlds (Lewis-Williams and Pearce 2004). If so, petroglyphs acted not as a picture on a wall, but as electrical sockets and circuits, points of access to a hidden network of power. The point is that much of Neolithic imagery may not have been imagery at all; it may have been a material operation on the world, important for what it was or for what the act of making it accomplished.

This is consistent with what we know of European Neolithic society. Many Neolithic groups show clear evidence for a preoccupation with ritual activities. From henges and “temples” to monumental tombs and complex processing of the dead, we can see a concern with understanding and intervening in the cosmos. At the same time, there is little convincing evidence anywhere for political or economic inequality. Building and using ritual structures may have required people in positions of authority, but such inequality as existed was probably restricted to the ritual sphere rather than involving generalised social control. This is typical of what Spielmann (2002) has called the “ritual mode of production”, in which ritual was an end in itself. It was a world in which (to coopt a phrase of Geertz (1980)), politics served ritual, not ritual politics. It may have been accompanied by a model of personhood which was situational or contextual, rather in the manner of so-called “Great Man” societies (Godelier and Strathern 1991): authority or capacity in one sphere of action did not necessarily convey power in other spheres (Fowler 2004; Robb and Harris 2018). In such a world, ritual knowledge can be both powerful and dangerous, and access to ritual knowledge may be unevenly distributed (Whitehouse 1992). Ritual knowledge may have been reproduced through practices which were contextually segregated if not downright secret, polysemic and encoded rather than superficial and transparent.

### The Bronze and Iron Ages: the Birth of Narrative Art

Things changed dramatically in the late 4th and 3rd millennia BC. Between 3500 BC and 2400 BC, in a transition beginning earlier in southern and eastern Europe and reaching north-western Europe later, there was a continent-wide reorganisation of society. As generations of archaeologists have noted, Bronze Age society differed profoundly from Neolithic society, with the 3rd millennium as a pivotal age of transition. Economically, people diversified from subsistence horticulture, with the widespread use of ploughing and with extended pastoralism as a way of colonising mountain landscapes. People mined, principally for exchangeable substances such as high-quality flint, copper and tin. Metals were important above all as a social valuable and a visually impressive body enhancement for both men and women. Both trade and new methods of transport (the horse, the sailed boat) meant that societies were more interconnected than ever before. The era of big ritual systems ended, and ritual as a whole was much less prominent; as a collective social project tying society and societies together, it may have been supplanted by trade systems. In many areas, collective burials were replaced by individual burials with grave goods displaying gender and status; even where collective burials persisted (as in Mediterranean collective tombs), burials were often deposited with status kits of individual goods.

These changes were clearly closely related within a basic social charter. What changed? Mass migration models have been discredited as over-simplistic; in every region of Europe, there is clear evidence for substantial continuity from the Neolithic. Technological and economic models correctly highlight the importance of metals and the “secondary products revolution” (Sherratt 1981), but tend to regard these as independent prime movers rather than as the consequences of human social choices. More to the point, social models have highlighted three key factors. First, Bronze Age society seems to have been much more gender-conscious; gender emerges as a key organising principal and an obligatory social category in contexts in which it often had not been marked before (Robb and Harris 2018). Secondly, in death rituals, Neolithic people emphasised generalised, possibly collective or anonymous ancestors; Bronze Age people thought in terms of genealogies anchored by specific forebears, perhaps reflecting a new emphasis on lineal descent (Barrett 1994; Thomas 1999). Finally, among the living, ritual was supplanted by prestige as a means of regulating society (Shennan 1982; Thorpe and Richards 1984). It is no accident that we see a coherent suite of symbols emerge, focused around ornaments for women and weapons for men. These form an interlocking group of displayable, transactable things which motivated and choreographed social interaction across diverse spheres of life (production, trade, display, deathways, art). It is also no accident that these things were used to identify and distinguish particular kinds of bodies. Obtaining, displaying and redistributing these items helped distinguish a new, gender-specific kind of social person, the generalised political leader.

These changes provide a context for the macro-changes in art identified here. In terms of location, Bronze and Iron Age art was placed in less particular or specified locations than previously. It may not have been made or used equally by all members of society, but it is less restricted. It is part of a discourse less segregated from ordinary life and groups, part of a more integrated discourse of social process. It is also more accessible cognitively. Its imagery contains more explicit information about what it is and how to interpret it, making it less dependent upon the viewer to supply context. It is more transparent, an art of surfaces rather than layers. And in adding detail to elements, and combining elements into groupings, it is increasingly about representation, about carrying a greater semantic burden than a single schematic motif can convey.

**Fig. 5:**
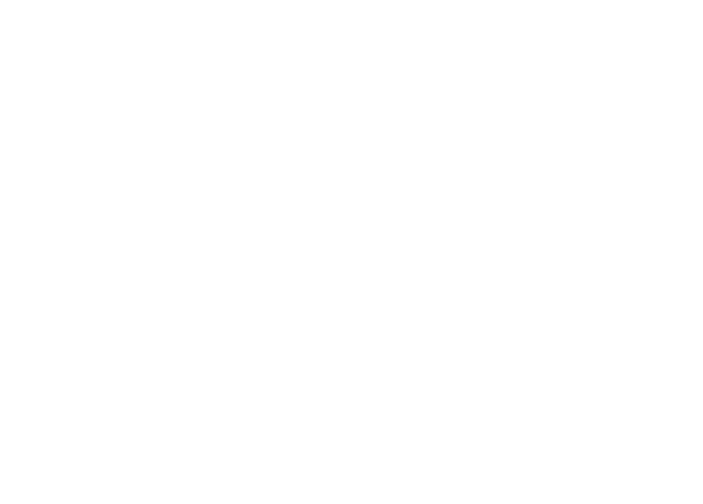
Actions in Bronze and Iron Age art. **a** Ploughing, Valcamonica, Italy (drawing: Vicki Herring). **b** Two warriors fighting, perhaps in ritual duel, Valcamonica, Italy (image: author). **c** Hunting, Valcamonica, Italy (image: author). **d** A ship filled with crew, Tanum, Sweden (drawing: Vicki Herring). **e** Two pairs of ithyphallic warriors fighting or dancing, Tanum, Sweden (redrawn after Coles 2005: Fig. 162). **f** Dance, Tanum, Sweden (image, author). **g** Acrobats, Tanum, Sweden (drawing: Vicki Herring). **h** Sexual activity, Svälte, Sweden (redrawn after Coles 2005: Fig. 55). **i** Horsemen riding, Thrace (redrawn after Pivalaki 2016: Fig. 14.2)

Above all, the Bronze Age sees the birth of narrative art. Archaeologists have often recognised a narrative element in Bronze and Iron Age art, either explicitly (Ranta et al. 2019) or implicitly by discussing the stories motifs may reference. What makes art narrative? Bronze and Iron Age art rarely includes explicit reference to sequential time (as in a comic-book style series of images, or the Chauvet Cave lion in which multiple images may show the same thing in a series of stop-action moments). But it is built out of recognisable elements which carry clear references to a broad audience (Ranta et al. 2019). Moreover, it often explicitly shows actions—people ploughing, warriors fighting, a boat loaded with crew and sailing, a rider mounted on a horse, a hunter chasing a quarry. Objects may also imply actions: a game animal implies hunting, a boat implies a journey and indeed a system of “maritime praxis” (Ling 2012). In some cases, they may have referenced specific narratives, much in the same way that any Christian image of Jesus references the narrative of his life, death and resurrection; this has been most explicitly suggested for a Bronze Age Scandinavian cosmological narrative (Bradley 2006; Kaul 1998). Above all, narrative art prompts the viewer to infer the nature of the connections between elements—a person on a horse is a hunter or a warrior, a boat full of people is on a journey, two armed people facing each other are having a duel, petroglyphs even have sex (Fig. 5). The images furnish hooks to hang stories upon. Viewing it re-orders your vision to see the world in terms of narratives of personhood.

What are these narratives about? They varied a lot—Bronze and Iron Age rock art depicts a huge range of things. But the major outlines show few surprises, converging well with what is known from archaeology. It is much more visibly gendered. There are a few gendered images in two-dimensional Neolithic imagery, but not many. In Bronze and Iron Age art, they abound. Gender may be shown anatomically, *via* conventional signs such as dot between the legs to indicate a woman, or *via* gendered objects and contexts. Much of it reflects male concerns. Indeed, it may have been made and used by gendered and aged sub-groups, perhaps to accompany story-telling (as with Comanche rock art (Fowles and Arterberry 2013)); there is no reason to suppose it reflects an accurate cross-section of society rather than sectional interests. But the themes are socially central ones.

Hunting art provides an apposite example. It is a rarely appreciated paradox of European prehistoric art that all imagery which shows the act of hunting itself—as opposed to simply the animals hunted and eaten—was actually made not by hunter-gatherers but by farmers. The earliest hunting art *per se* seems to be one panel at Porto Badisco, Italy, which dates to the later Neolithic; there are similar scenes in Iberian Schematic Art. Hunting art becomes much more frequent in both Scandinavian and Alpine Bronze-Iron Age rock art; there are also representations in contemporary media such as Mycenaean metalwork and the Daunian stelae which suggest it represents a Europe-wide genre of action. Why? It has nothing to do with subsistence; all of these groups lived on crops and domesticated animals. Instead, perhaps expressly because hunting was superfluous to subsistence, hunting became a prestigious activity, a social drama of individual maleness and status—which was not only done, but narrated.

The most prominent example of visual narrative, warfare, shows how imagery echoes what we see in traded and displayed goods and in burials. Archaeologists have noted the Bronze Age origin of the warrior as a new kind of social figure (Harding 2007; Guilaine and Zammit 2005); this is based above all on how weapons are used to define personas in burial, but weapons also figure as important items of material culture among the living. Bronze and Iron Age rock art is full of warriors. Weapons make their appearance in Copper Age imagery at Valcamonica and Mount Bego, occasionally with people holding them. From the Bronze Age onwards, in southern Scandinavia, beweaponed men are common, and boats loaded with men probably reference armed raids. Armed figures in Iron Age Valcamonica often occur in pairs fighting what may have been understood as ritual duels. It is also a common theme in inset scenes on Iberian Bronze Age stelae, Iron Age situlae, and even Greek Geometric pottery. We do not know whether such images referenced real people and their histories, or figures of myth or legend, or indeed whether the images formed part of some visual speech act—a declaration of prowess, a prayer for an outcome. But in all these cases, in visual narratives, the warrior has arrived. Indeed, as Ling and Cornell (2010) have argued powerfully, such visual narratives may have been the means by which warriors were produced.

## Conclusions: Sketching art’s (Pre)History on a Broad Canvas

Prehistoric Europe’s visual culture travelled a long road between 6000 and 0 BC. The Neolithic inspires a deep feeling of alterity. There is an unfamiliar logic at work which often refuses to make sense to us. It is telling that comparative models for the Bronze Age tend to look forward to historic times, to the threshold of the Classical period; comparative models for the Neolithic tend to look elsewhere, to “tribal” worlds in the Americas, in Africa, in Melanesia. This is as true for visual culture as for other aspects of life. Not only do we usually not know what Neolithic “art” depicts, we often do not even know how to interpret it. This is not simply due to our ignorance of a prehistoric denotative code. The carvings and paintings themselves are schematic, perhaps intentionally obscure, perhaps polysemic, perhaps restricted (Bradley 1997). It may represent encoded ritual knowledge with little concern to be understood by non-practitioners; it may not have been intended to represent anything, but rather to do something, to perform some ritual action or to provide the means for doing so. In any case, it is not pictures as we know them.

The continent-wide revolution of the 3rd millennium BC reformulated European society along new principles of social reproduction. Not surprisingly, visual culture changed too. Indeed, it is striking that nowhere in Europe can we identify a single specific body of art which spans the two periods continuously, something which makes it difficult to highlight such trends based upon studies of single corpora alone, but which also underlines the broad change in how art worked and what it was used for. The nearest we can find are occasional bodies of imagery which include characteristics of both periods (e.g. Monte Bego, the Copper Age component at Valcamonica). On a thematic level, it reflects new concerns: gender, economic production, hunting, warfare. As visual culture, it works differently, with more detail and more grouped images tying in to narratives about protagonists and their situations. It is also more transparently representational. Representational narrative art created a new way of seeing (*sensu* Berger 1972). And this tied into a new social role for imagery. By the Iron Age, it increasingly resembles pictures as we understand them: visual signs which represent narratives, increasingly narratives of personhood populated with social types known from other contexts.

Explicitly or implicitly, art histories work from and against the baseline of a big history which brings major forms of visual culture together into a continuous narrative. In any study of a particular artist’s oeuvre, writer and reader both already know what makes a work “Classical” or “Medieval”, “Impressionist” or “Modernist”. Indeed, such knowledge forms the background for understanding the unique features of each artist or school and the ways their works must be viewed. For prehistoric visual culture, we have never had such a history. This article aims to show that such a history is both possible and useful. Here, I have discussed only two-dimensional imagery, leaving aside 3D works such as figurines and sculptures, which may have followed their own pathways. Both the patterns I identify and the interpretations I give them are simple and obvious in retrospect. Prehistorians have long acknowledged that Neolithic art works differently than we expect it to and have related this to the alterity of Neolithic society. Similarly, they have responded, usually implicitly, to the comprehensible style of transparent imagery and narrative reference of Bronze and Iron Age art, and the fact that the narratives involved reflect the gendered prestige and politics of the time. The provocative element of this paper is simply posing the question of how the two periods relate as part of a general history, a project prehistorians have never really conceived of, much less tackled seriously. As a first attempt, it is tentative, and future work will no doubt prove some aspects incorrect. But if it inspires future work, even to contradict it, it will have accomplished its goal. The potential is great. Here I explore merely one moment in such a history, albeit a central one; much as histories of art have built progressively outwards over time from a focus upon the medieval-to-Renaissance transition, the transition from Neolithic to Bronze Age art can anchor a global prehistory of art yet to be developed. Such a history would encompass periods of alterity such as Palaeolithic and Mesolithic art and the fascinating world of Iron Age to early medieval art in Northern Europe, different genres—how does three-dimensional art tell the same or different stories?—and relations with alternative traditions such as circum-Arctic, North African and Near Eastern palaeo-art.

## Footnotes

1 One could make a plausible argument that Scythian metalwork and the later Iron Age La Tène metalwork style, also called “Celtic” art, also represent the start of a different macro-tradition whose subtle and inextricable mixing of animal and geometric designs had ontological implications and which developed seamlessly into “animal art” and other genres of early medieval Northern Europe (Gosden et al. 2008; Garrow and Gosden 2012).
